# Effects of interleukin-1 receptor antagonism in women with polycystic ovary syndrome—the FertIL trial

**DOI:** 10.3389/fendo.2024.1435698

**Published:** 2024-09-11

**Authors:** Milica Wälchli-Popovic, Sophie Monnerat, Angela E. Taylor, Lorna C. Gilligan, Lina Schiffer, Wiebke Arlt, Deborah R. Vogt, Christian De Geyter, Nina Hutter, Marc Y. Donath, Gideon Sartorius, Mirjam Christ-Crain

**Affiliations:** ^1^ Department of Endocrinology, Diabetology and Metabolism, University Hospital Basel, Basel, Switzerland; ^2^ Department of Clinical Research, University of Basel, Basel, Switzerland; ^3^ Institute of Metabolism and Systems Research, University of Birmingham, Birmingham, United Kingdom; ^4^ Desai Sethi Urology Institute, Sylvester Comprehensive Cancer Center, University of Miami, Miami, FL, United States; ^5^ Medical Research Council Laboratory of Medical Sciences (MRC LMS), Institute of Clinical Sciences, Faculty of Medicine, Imperial College London, London, United Kingdom; ^6^ Institute of Clinical Sciences, Faculty of Medicine, Imperial College London, London, United Kingdom; ^7^ Reproductive Medicine and Gynecological Endocrinology (RME), University Hospital, University of Basel, Basel, Switzerland; ^8^ Fertisuisse, Olten/Basel, Switzerland

**Keywords:** interleukin-1, PCOS, polycystic ovary syndrome, hyperandrogenemia, anakinra, inflammation

## Abstract

**Introduction:**

Chronic low-grade inflammation might contribute to hyperandrogenemia and metabolic complications in polycystic ovary syndrome (PCOS). The proinflammatory cytokine interleukin (IL)-1 stimulates androgen production from ovarian cells, whereas blockade of the IL-1 pathway improves cardiometabolic health. We aimed to investigate whether blocking the IL-1 pathway ameliorates hyperandrogenemia in patients with PCOS.

**Methods:**

This is a prospective, interventional, single-arm, proof-of-concept trial performed at a tertiary hospital in Switzerland (August 2018 to July 2020) in 18 premenopausal women with a diagnosis of PCOS according to the Rotterdam criteria, total testosterone levels ≥ 1.7 nmol/L, and C-reactive protein (CRP) ≥ 1.0 mg/L. Patients received 100 mg/day of the IL-1-receptor antagonist anakinra for 28 days and underwent weekly blood sampling until 1 week after the end of treatment. The primary endpoint was the change in serum androstenedione levels on day 7 of treatment, assessed with liquid chromatography–tandem mass spectrometry. Seven of these women participated in a subsequent observational sub-study (May 2021 to December 2021).

**Results:**

Median [interquartile range (IQR)] androstenedione increased by 0.5 [−0.1, 1.6] nmol/L (*p* = 0.048) with anakinra and by 1.3 [0.08, 2.4] nmol/L [*p* = 0.38] without anakinra between baseline and day 7. Anakinra reduced CRP levels on days 7, 21, and 28 (*p* < 0.001) but did not lead to an absolute reduction in androgens. However, four of six patients (67%) had smaller areas under the curves for androstenedione and/or testosterone during the 28-day intervention with anakinra as compared to 28 days without treatment.

**Discussion:**

Our findings suggest that anakinra suppresses IL-1-mediated chronic low-grade inflammation in PCOS and might attenuate biochemical hyperandrogenemia.

## 1 Introduction

Polycystic ovary syndrome (PCOS) is a common endocrine disorder in women of child-bearing age, presenting with reproductive (i.e., hyperandrogenism, oligo-, or amenorrhea) as well as metabolic features (e.g., insulin resistance and cardiovascular risk) (1–6). Despite the high prevalence of PCOS, its underlying causes are still unknown, which has substantially limited the development of causative treatments (7, 8). Although PCOS is a heterogeneous and multifaceted disorder, the current therapeutic approaches only focus on single symptomatic aspects disregarding other PCOS-related and/or metabolic problems (9–11). Thus, investigating pathophysiologic mechanisms in women with PCOS could provide new therapeutic targets for a holistic approach to PCOS, which is desperately needed (12).

In the past 20 years, numerous observational studies have shown an association between chronic low-grade inflammation and PCOS (13). A meta-analysis revealed that women with PCOS had elevated C-reactive protein (CRP) levels compared with weight-matched controls (10). It has been shown to be associated with central fat excess, and thus more with fat distribution pattern than with increased fat mass *per se* (14). CRP is the most useful clinical marker for chronic low-grade inflammation and mirrors interleukin (IL)-1β activity (15). IL-1β is secreted by peripheral mononuclear cells upon inflammasome activation by proinflammatory stimuli (e.g., glucose, reactive oxygen species, and saturated fat) (16–18). Chronic IL-1β activity has deleterious effects on glucose metabolism as well as cardiovascular health (19, 20).

Importantly, the capability of IL-1β to trigger and increase androgen production by theca cells via activation of the inflammasome and upregulation of steroidogenesis was recently demonstrated *in vitro* (21). Further experimental data showed that IL-1β as well as IL-1α, another ligand of the IL-1 receptor, disturbs regular steroidogenesis and subsequent follicle maturation and leads to a decrease in fertility rates (22). Several clinical studies indicated a clear association between inflammatory markers and serum androgens (18). Moreover, macrophages in the ovaries from women with PCOS show a predominantly proinflammatory phenotype (23). An interventional study investigating anti-inflammatory treatment with the NFκB inhibitor salsalate in eight women with PCOS demonstrated improvement in ovarian steroid metabolism and suggested positive effects on ovulatory function (24).

We therefore hypothesized that IL-1 might be causally linked to hyperandrogenemia in women with PCOS and aimed to investigate the effect of IL-1 pathway blockade with anakinra on androgen levels in women with PCOS.

## 2 Materials and methods

### 2.1 Study design and participants

This investigation consists of two studies, a main interventional study and a subsequent observational sub-study. The main interventional study was designed as a single-arm trial without placebo because of the available resources. We performed a follow-up observational sub-study without any treatment in a subset of the same cohort; this was initiated and conducted following completion and data analysis of the main interventional study.

The first and main study was a prospective, interventional, open-label, proof-of-concept single-arm trial conducted at the University Hospital Basel in Switzerland. Eighteen premenopausal women with a diagnosis of PCOS according to the Rotterdam criteria and biochemically confirmed hyperandrogenemia and low-grade inflammation were recruited from August 2018 until June 2020 and were treated with 100 mg of the IL-1 receptor antagonist anakinra (Kineret^®^) by subcutaneous injections once daily for 4 weeks. The 100-mg daily dose was chosen because it is the standard dose used for its commonest indication rheumatoid arthritis for which most data on safety, pharmacokinetics, and pharmacodynamics are available (25, 26). The following sub-study was a prospective observational study conducted at the University Hospital Basel in Switzerland from May 2021 to December 2021. Seven of 18 (39%) patients who participated in the main trial agreed to participate in the sub-study, during which they underwent weekly blood samplings for 5 weeks without receiving any study intervention. The conduct of both studies adhered to the Declaration of Helsinki and Good Clinical Practice guidelines. The local ethics committee (EKNZ-2018-00780 and EKNZ-2021-00607) and Swissmedic (2018DR2072) approved the clinical studies before starting patient recruitment. All patients provided written informed consent. The interventional phase was registered on ClinicalTrials.gov (NCT03578497).

**Figure 1:**
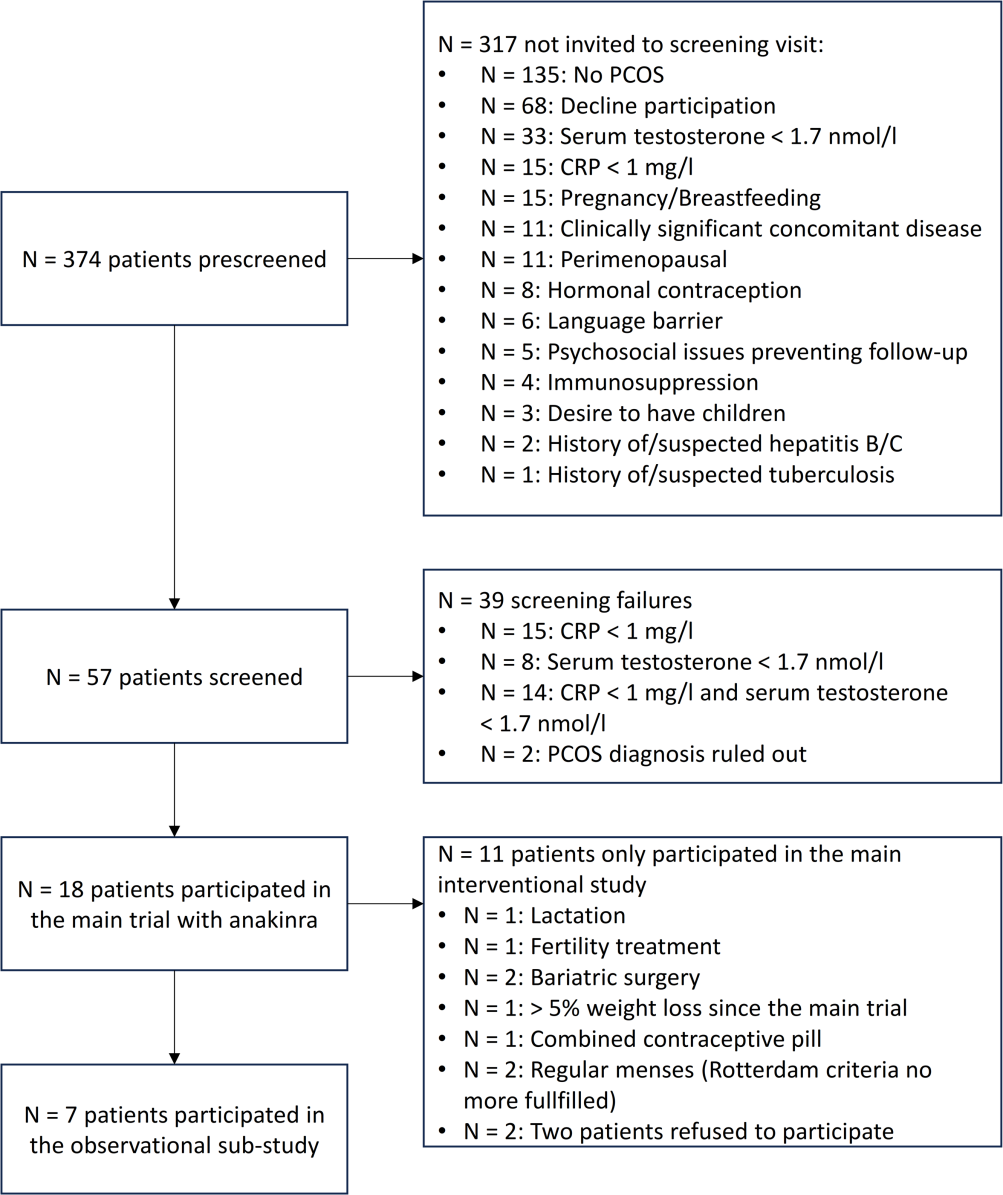
Study flowchart. CRP, C-reactive protein; PCOS, polycystic ovary syndrome.

The study included adult, premenopausal women with (i) a diagnosis of PCOS according to the Rotterdam criteria (27), (ii) hyperandrogenemia in routine assessment (total testosterone levels ≥ 1.7 nmol/L, measured with an electrochemiluminescence immunoassay), and (iii) low-grade inflammation (CRP ≥ 1.0 mg/L). Main exclusion criteria were hormonal treatments (e.g., contraceptives) in the 2 months prior to the investigational phase, pregnancy or breastfeeding, contraindications to anakinra, clinical signs of infection, treatment with immunosuppressive drugs, severe concomitant diseases, or a history of suspected or confirmed tuberculosis and/or hepatitis (**Figure 1**). Additional exclusion criteria of the observational sub-study included a loss of >5% of body weight since the end of the first trial and hormonal treatment for at least 8 weeks prior to study start (except for gestagen treatment to induce withdrawal bleedings) (**Figure 1**).

### 2.2 Study procedures

The interventional study consisted of eight study visits at the University Hospital Basel. Blood sampling was performed at each visit between 7:30 and 10:00 a.m. after an overnight fast, and pregnancy was ruled out before inclusion. Treatment start was only possible in the follicular phase of the menstrual cycle, which was ensured by scheduling the baseline visit on days 3–5 of either gestagen-induced withdrawal bleeding or spontaneous onset of menstruation. Alternatively, if patients refused gestagen intake, cycle phase was determined based on the constellation of baseline estradiol, progesterone, follicle-stimulating hormone (FSH), and luteinizing hormone (LH) levels. Baseline assessment included a PCOS-specific medical history and clinical examination. Included patients received one package with 28 syringes each containing 100 mg of anakinra and were instructed for daily subcutaneous application. In addition, patients were advised not to start a new lifestyle or drug treatments during study time. Patients then came weekly until the end of treatment with anakinra (visit 5 on day 28).

The observational sub-study consisted of five study visits at the University Hospital Basel. Blood samplings were scheduled between 7:30 and 10:00 a.m. after an overnight fast and pregnancy was ruled out before inclusion. Study start was only possible in the follicular phase of the menstrual cycle, which was ensured by spontaneous onset of menstruation or was determined based on the constellation of baseline estradiol, progesterone, FSH, and LH levels. Included patients were asked to come for a weekly visit over the four following weeks.

### 2.3 Study endpoints

The primary objective was to investigate whether the IL-1 receptor antagonist anakinra ameliorates hyperandrogenemia in women with PCOS. Secondary objectives included the effect of the IL-1 receptor antagonist on inflammation, menstrual bleedings, clinical hyperandrogenism, and other laboratory parameters.

The primary outcome was the absolute change in fasting serum androstenedione levels from baseline to day 7 with and without anakinra. Secondary endpoints included changes in serum androstenedione and serum testosterone on days 7, 14, 21, and 28 with and without anakinra and 1 week after the end of treatment with anakinra. In addition, the course of further laboratory parameters [serum anti-Mullerian hormone (AMH), estradiol, progesterone, LH, FSH, and CRP levels] was described and investigated as predictors for changes in androgen levels and the onset of menstruation. Furthermore, the effect of anakinra on changes in additional androgens [11β-hydroxyandrostenedione (11OHA4), 11β-hydroxytestosterone (11OHT), 11-ketoandrostenedione (11KA4), 11-ketotestosterone (11KT), dehydroepiandrosterone (DHEA), and dehydroepiandrosterone sulfate (DHEAS)] and the role of the predominant origin of androgens (ovarian or adrenal) were presented. Changes in acne scores were evaluated on day 28 of treatment with anakinra based on the Plewig–Kligman score (27).

### 2.4 Laboratory measurements and sample handling

To assess hyperandrogenemia for study inclusion, serum total testosterone levels were measured with an electrochemiluminescence immunoassay (ECLIA, Elecsys Testosterone II, Roche Diagnostics GmbH). AMH and estradiol levels were quantified with an ECLIA (Elecsys AMH Plus, and Elecsys Estradiol III, Roche Diagnostics GmbH). After blood coagulation, serum tubes were centrifuged at −4°C, 3000 rpm for 10 min and stored at −76°C. Serum androgens (androstenedione and total testosterone) and progesterone were measured by a multi-steroid profiling liquid chromatography tandem-mass spectrometry (LC-MS/MS) method at the University of Birmingham Steroid Metabolome Analysis Core, as previously described (28, 29). FSH, LH, SHBG, and baseline progesterone were measured with ECLIA (Elecsys FSH, Elecsys LH, Elecsys SHBG, Roche Diagnostics GmbH).

### 2.5 Power calculation

The assumed effect size and standard deviation were based on the work of Jensterle et al. (30) who report a mean reduction in serum androstenedione levels of 2.7 nmol/L after 12 weeks of treatment with metformin and standard deviations at baseline and at the end of treatment of 3.7 and 3.3 nmol/L, respectively. Assuming an intermediate within-patient correlation of 0.6, we derived a standard deviation of 3.15 nmol/L. Using a two-sided one-sample *t*-test, we estimated that a sample size of 18 patients would provide 93% power for an assumed effect size of 2.7 mmol/L reduction in androstenedione and 80% power for a reduction of 2.2 mmol/L to reject the null hypothesis of no change at a significance level *α* of 0.05.

### 2.6 Statistical analysis

All analyses were performed using the statistical program R (version 4.2.3 or higher). Baseline characteristics are summarized using descriptive statistics. A two-sided significance level of 0.05 was used for every analyses except for interaction terms of linear mixed models for which we considered a *p*-value < 0.1 as justification for subgroup analyses. *p*-values were not adjusted for multiple testing.

The primary endpoint, the change in fasting serum androstenedione level from baseline to day 7, was tested with a paired Wilcoxon signed-rank test. Subgroup effects for the primary endpoint were evaluated in the interventional dataset only, by fitting linear regression models with log-transformed androgen values during treatment as outcome variable, baseline androgen values as covariate, and subgroup and their interaction term with the covariate as predictors.

Androstenedione, testosterone, and CRP values at each visit were compared to baseline values with a paired Wilcoxon signed-rank test. The area under the curve (AUC) of changes in androgens between day 0 and day 28 with and without anakinra was computed for each patient who participated in the interventional and in the observational study. Additional androgens (11OHA4, 11OHT, 11KA4, 11KT, DHEA, and DHEAS) were compared graphically with boxplots. The predominant origin of androgens (ovarian or adrenal) was evaluated dividing baseline androstenedione by 11OHA4 and comparing the participants with a quotient below versus above the median.

A more detailed description of the statistical methodology can be found in the **Supplementary Materials**.

## 3 Results

### 3.1 Interventional study with anakinra

#### 3.1.1 Baseline characteristics

Baseline characteristics of the investigational study (*n* = 18) are shown in **Supplementary Table S1**. Median [interquartile range (IQR)] age was 27 [23, 32] years and median [IQR] body mass index (BMI) was 32.6 [28.8, 37.3] kg/m^2^. Eighty-nine percent of the patients reported either oligo- or amenorrhea. Polycystic ovary morphology was present in 78% of the patients. Baseline prolactin and 17-OH progesterone were in the normal range, 322 mlU/L [203, 387] and 3.4 nmol/L [2.1, 4.2], respectively. Median [IQR] hirsutism (Ferriman-Gallwey) score was 7 [1, 12]. One patient (6%) suffered from alopecia, and three patients (17%) presented with acanthosis nigricans. Varying degrees of acne were present in 72% (*n* = 13/18) of the patients. Out of the eight patients who had tried to conceive, five (63%) had a history of infertility and four (50%) had a history of miscarriage. Four patients (22%) had concomitant treatment with metformin.

One patient withdrew consent after day 7 and, therefore, was only included in the primary endpoint analysis.

#### 3.1.2 Effects of IL-1 receptor antagonism on androgen levels

During the interventional study (*n* = 18), median [IQR] androstenedione and testosterone levels were 7.1 [5.8, 8.1] nmol/L and 1.4 [1.2, 1.7] nmol/L at baseline, respectively. The respective median [IQR] changes from baseline to day 7 were 0.5 [−0.1, 1.6] nmol/L (*p* = 0.048) and 0.2 [0.0, 0.3] nmol/L (*p* = 0.04). The minimal and maximal changes in androstenedione were −5.8 and 3.8 nmol/L. Serum androstenedione or testosterone concentrations on days 14, 21, 28, and 35 did not differ significantly from baseline levels (**Table 1**, **Supplementary Figures S1**, **S2**). In summary, there was overall no absolute reduction in androgens upon anakinra. Amenorrhea at baseline (*p* = 0.03) but not baseline CRP (*p* = 0.53), CRP reduction (*p* = 0.53), the presence of injection site reaction (*p* = 0.57), or the onset of menstruation (*p* = 0.13) or ovulation (*p* = 0.79) was a predictor for changes in androstenedione. The change in androstenedione between baseline and day 7 was 0.36 [0.02, 1.20] nmol/L in patients with amenorrhea at baseline as compared to 0.69 [−0.03, 1.6] nmol/L in patients with oligo-/normomenorrhea (*p* = 0.17).

**Table 1:** Changes in laboratory values upon anakinra.

Study time	Baseline	Day 7	*p*	Day 14	*p*	Day 21	*p*	Day 28	*p*	Day 35	*p*
*n*	18	18		17		17		17		17	
Androstenedione, nmol/L	7.1 [5.8, 8.8]	7.8 [6.3, 10.6]	*0.048*	7.5 [5.8, 9.4]	*0.48*	7.1 [6.1, 9.9]	*0.71*	7.1 [5.9, 9.9]	*0.91*	7.6 [6.2, 9.6]	*0.35*
Testosterone, nmol/L	1.4 [1.2, 1.7]	1.5 [1.3, 2.1]	*0.04*	1.5 [1.2, 1.8]	*0.21*	1.6 [1.2, 2.0]	*0.39*	1.4 [1.2, 2.0]	*0.41*	1.4 [1.1, 1.8]	*0.94*
CRP, mg/dL	3.3 [1.3, 5]	0.7 [0.5, 1.2]	*<0.001*	1.2 [0.7, 6.1]	*0.13*	1.1 [0.7, 2.4]	*<0.01*	1.1 [0.8, 1.3]	*<0.001*	3.1 [1.5, 4.5]	*0.66*
Oestradiol, pmol/L	191 [164, 225]	209 [201, 245]	*0.01*	208 [195, 301]	*0.01*	214 [181, 305]	*0.01*	204 [158, 225]	*0.13*	*194 [158, 225]*	*0.10*
Progesterone, nmol/L	0.27 [0.18, 0.40]	0.19 [0.12, 0.35]	*0.30*	0.35 [0.20, 0.47]	*0.55*	0.26 [0.18, 0.43]	*0.78*	0.35 [0.16, 0.42]	*0.51*	0.29 [0.20, 0.42]	*0.59*
FSH, IU/L	5.8 [4.8, 6.3]	6.1 [4.7, 6.9]	*0.98*	5.7 [5.0, 6.5]	*0.74*	6.0 [2.9, 6.4]	*0.60*	5.7 [4.6, 6.4]	*0.32*	*5.4 [4.5, 5.9]*	*0.18*
LH, IU/L	13.8 [11.4, 18.5]	14.1 [10.9, 18.0]	*0.85*	15.6 [10.5, 20.6]	*0.52*	14.1 [9.4, 16.8]	*0.44*	11.8 [9.0, 18.7]	*0.62*	*12.3 [9.4, 18.5]*	*0.94*
AMH, pmol/L	55.2 [49.5, 88.3]	58.3 [49.5, 82.5]	*0.12*	58.0 [46.3, 72.4]	*0.26*	62.2 [46.7, 81.2]	*0.46*	56.6 [44.9, 85.7]	*0.04*	*54.5 [39, 69.5]*	*0.94*
SHBG, nmol/L	27.1 [17.0, 39.6]	29.2 [16.3, 41.6]	*0.02*	23.4 [16.5, 38.8]	*0.96*	27.3 [16.2, 43.6]	*0.14*	24.6 [15.7, 44.2]	*0.31*		

Serum androgens were measured with tandem mass spectrometry (LC-MS/MS). High-sensitivity CRP was measured with an immunoturbidimetric assay (Tina-quant C-Reactive Protein IV; Roche Diagnostics GmbH). AMH, estradiol, FSH, LH, and SHBG were measured with ECLIA (Elecsys AMH Plus, Elecsys Estradiol III, Elecsys FSH, Elecsys LH, and Elecsys SHBG, Roche Diagnostics GmbH).

**Figure 2:**
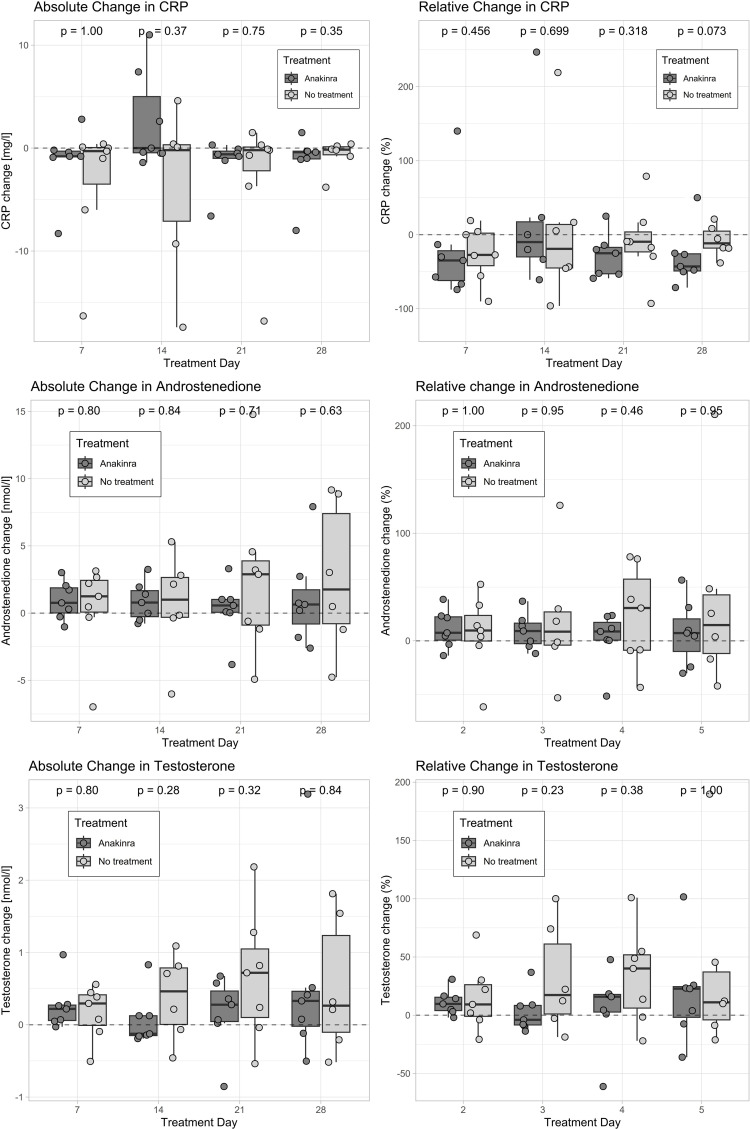
Change in androstenedione, testosterone, and CRP with and without anakinra. Only patients who participated in both the interventional and the observational studies are displayed (*n* = 7). Absolute changes were computed by subtracting the baseline concentration from the corresponding follow-up value. Values above the dashed line represent an increase and values below the dashed line represent a decrease as compared to baseline. Androgens were measured with tandem-mass spectrometry (LC-MS/MS), and CRP was measured with an immunoturbidimetric assay (Tina-quant C-Reactive Protein IV; Roche Diagnostics GmbH). CRP, C-reactive protein.

Serum androstenedione and testosterone concentrations during anakinra treatment in the subset of patients who also participated in the subsequent observational phase can be found in **Figure 2**, **Table 2**, and **Supplementary Table S2**.

**Table 2:** Absolute and relative change from baseline in androgens and CRP.

Study day	Day 7			Day 14			Day 21			Day 28		
Treatment	Anakinra	Anakinra	No treatment	Anakinra	Anakinra	No treatment	Anakinra	Anakinra	No treatment	Anakinra	Anakinra	No treatment
*n*	18	7	7	17	7	6	17	7	7	17	7	6
Absolute change in androstenedione, nmol/L	0.5 [−0.1, 1.6]	0.77 [0.0, 1.9]	1.3 [0.1, 2.4]	0.3 [0.0, 1.3]	0.8 [−0.3, 1.7]	1.0 [−0.3, 2.7]	0.5 [−0.1, 1.0]	0.6 [0.06, 1.0]	2.9 [−0.9, 3.9]	0.2 [−1.2, 0.7]	0.6 [−0.8, 1.7]	1.8 [−0.8, 7.4]
Relative change in androstenedione, %	7.1 [−1.3, 22.3]	7.4 [0.7, 22.3]	9.6 [−0.2, 23.6]	6.7 [−0.2, 13.9]	9.1 [−2.6, 16.4]	8.5 [−4.0, 26.8]	8.7 [−0.8, 19.0]	8.7 [0.6, 17.1]	30.5 [−8.7, 57.4]	4.3 [−15.8, 9.8]	7.2 [−9.9, 20.4]	14.7 [−11.8, 42.7]
Absolute change in testosterone, nmol/L	0.2 [0.0, 0.3]	0.2 [0.1, 0.3]	0.3 [0.0, 0.4]	0.1 [−0.1, 0.1]	−0.1 [−0.2, 0.1]	0.5 [0.0, 0.8]	0.1 [0.0, 0.4]	0.3 [0.05, 0.5]	0.7 [0.1, 1.1]	0.1 [−0.1, 0.3]	0.3 [−0.02, 0.5]	0.3 [−0.1, 1.2]
Relative change in testosterone, %	11.6 [1.2, 23.3]	9.7 [4.0, 15.4]	9.3 [−1.0, 26.2]	7.9 [−7.9, 17.9]	−4.0 [−8.4, 8.4]	17.3 [1.0, 61.1]	10.7 [−0.3, 20.3]	15.9 [2.7, 17.8]	40.1 [6.1, 51.8]	3.9 [−10.0, 22.8]	22.8 [−1.8, 24.6]	11.03 [−4.0, 37.1]
Absolute change in CRP, mg/L	−1.8 [−4.3, −0.5]	−0.8 [−0.9, −0.3]	−0.3 [−3.5, 0.1]	−0.4 [−3.2, 2.6]	0.0 [−0.5, 5.0]	−0.2 [−7.1, 0.3]	−0.8 [−4.0, −0.5]	−0.6 [−1.0, −0.3]	−0.2 [−2.2, 0.1]	−0.7 [−3.0, −0.3]	−0.4 [−1.1, −0.3]	−0.2 [−0.7, 0.1]
Relative change in CRP, %	−70.4 [−85.9, −35.2]	−34.8 [−61.9, −21.7]	−27.3 [−41.8, 2.1]	−20.0 [−75.0, 37.1]	0.0 [−26.7, 134.9]	−19.1 [−45.0, 13.8]	−50.0 [−68.9, −17.1]	−25.0 [−52.8, −17.1]	−9.5 [−23.2, 3.8]	−42.9 [−71.4, −25.0]	−42.9 [−48.9, −25.8]	−11.7 [−18.1, 4.9]

Changes are relative to the baseline value of each parameter. Values are represented as median and interquartile range. Androgens were measured with tandem-mass spectrometry (LC-MS/MS), and CRP was measured with immunoturbidimetric assay (Tina-quant C-Reactive Protein IV; Roche Diagnostics GmbH).

CRP, C-reactive protein.

#### 3.1.3 Effects of IL-1 receptor antagonism on CRP levels

During the interventional study, median [IQR] CRP levels were 3.3 [1.3–5] mg/L at baseline and decreased to 0.7 [0.5–1.2] mg/L after 7 days of treatment with anakinra. At day 14, CRP reduction was counteracted by injection site reactions (median [IQR]: 1.2 [0.7–6.1] mg/L). Injection site reactions had mostly regressed by day 28, whereby CRP levels decreased to 1.1 [0.8–1.3] mg/L. One week after treatment discontinuation (day 35), CRP values increased to 3.1 [1.5–4.5] mg/L. Absolute and relative changes in CRP as well as subgroup analyses in women who participated in both study phases can be found in **Figure 2**, **Table 2**, and **Supplementary Table S2**.

#### 3.1.4 Effects of IL-1 receptor antagonism on the pituitary–ovarian axis, menstruation, and clinical hyperandrogenism

During treatment with anakinra, median estradiol levels were significantly higher on days 7, 14, and 21 as compared to baseline, an effect that was largely driven by patients who later reported menstrual bleeding. The detailed course of serum estradiol, FSH, LH, and AMH is shown in **Table 1**, **Supplementary Table S2**, and **Supplementary Figure S4**.

Three patients displayed a progesterone peak above the cutoff of 5.8 nmol/L for ovulation and one patient showed a progesterone peak at 4.97 nmol/L (**Supplementary Figure S3**). All of them were considered to have ovulated and experienced subsequent menstrual bleedings. Two of these four patients took metformin. One more patient, i.e., five patients in total, experienced a regular menstrual bleeding that started on treatment day 28 with anakinra in four of the patients. The fifth patient reported menstruation closely after termination of the study. Three of these five patients were oligo- and/or amenorrhoeic before the study started. The other two patients had regular cycles of 30 and 31 days.

There was no indication towards a change in sebum production or acne scores after the end of treatment with anakinra.

#### 3.1.5 Compliance and adverse events

At day 14, all patients (*n* = 17) had injection site reactions (7 mild, 6 moderate, and 4 severe), which mostly started on the ninth day of injection. A concurrent increase in CRP was observed (**Tables 1**, **2**, **Supplementary Figure S2**). In total, 12 patients (67%) opted for rescue antihistaminic treatment. On day 28, injection site reactions were partly resolved (*n* = 13) or fully resolved (*n* = 4). In addition, four patients reported upper respiratory tract symptoms during study time and one patient developed flu-like symptoms (fever and malaise) on the last day of treatment. The other adverse events were considered unrelated to the study drug.

Compliance was ensured by injection documentation in a study diary, and IL-1 receptor antagonist levels that were in the pharmacological range in all patients on treatment on days 7, 14, 21, and 28 were measured (data not shown). Thirteen patients took all 28 doses, four patients took 27 of 28 doses, and one patient withdrew consent on day 10 after taking 8 of 10 doses.

### 3.2 Observational sub-study without anakinra

#### 3.2.1 Baseline characteristics

Baseline characteristics at the start of the observational phase can be found in the **Supplementary Materials** (**Supplementary Table S1**). Of note, one patient had recently started intake of 100 mg of oral progesterone daily. The observation period started on the third day of her withdrawal bleeding and she was asked to restart progesterone 14 days after the onset of bleeding.

#### 3.2.2 Natural course of androgen levels without anakinra

No change was observed in serum androstenedione and testosterone in the observational study without anakinra (**Figure 2**, **Table 2**, **Supplementary Table S2**). Median [IQR] baseline androstenedione levels were 11.4 [8.3, 12.4] nmol/L and increased by 1.3 [0.08, 2.4] nmol/L (*p* = 0.38), while testosterone levels were 2.1 [1.9, 2.9] and increased by 0.3 [−0.01, 0.4] nmol/L (*p* = 0.38) after 7 days.

#### 3.2.3 Pituitary–ovarian axis and menstruation without anakinra

In addition to the patient taking oral progesterone, a progesterone peak was observed in one patient in whom the probable subsequent menses could not be recorded. Two more patients experienced menses without progesterone peak during the observational phase without anakinra. One of these menstruating patients had 10 menstruations and the other patient had 6 menstruations in the 12 months preceding the observational phase. Further changes in the pituitary–ovarian axis are summarized in **Supplementary Table S2**.

### 3.3 Comparison of androgen levels with and without anakinra

On day 7, four patients (57%) had a greater absolute increase in androstenedione whereas four patients (57%) had a smaller increase in testosterone with anakinra than without any intervention. Two patients had a reduction of both androgens, two patients had an increase in both, two patients had only a reduction in testosterone, and one patient had only a reduction in androstenedione.

AUCs of changes in androgens between baseline and day 28 with and without anakinra are displayed for six patients who participated in the interventional and observational studies in **Figure 2** and **Table 3** (the one patient taking progesterone during the observational study was excluded). Both the AUC for serum androstenedione and testosterone were smaller during anakinra use in three (50%) of the six patients. One patient (17%) had a smaller AUC of androstenedione but a larger AUC of testosterone with anakinra, one patient (17%) had the opposite constellation, and one patient had an increase in both AUCs.

**Table 3:** Area under the curve for changes in androgens with and without anakinra.

Patient	Androstenedione						Testosterone						Weight change		
	AUC with anakinra (nmol/L)	AUC without anakinra (nmol/L)	Absolute AUC difference (nmol/L)	Change to day 7 with anakinra (nmol/L)	Change to day 7 without anakinra (nmol/L)	Difference in change to day 7 (nmol/L)	AUC with anakinra (nmol/L)	AUC without anakinra (nmol/L)	Absolute AUC difference (nmol/L)	Change to day 7 with anakinra (nmol/L)	Change to day 7 without anakinra (nmol/L)	Difference in change to day 7 (nmol/L)	Absolute difference in weight (kg)		Absolute difference in BMI (kg/m^2^)
1	0.1	−12.0	+12.1	0.3	−0.3	+0.6	1.2	5.3	−4.1	0.3	0.4	−0.1	+8.0		+3.1
2	17.3	205.9	−188.6	3.0	2.7	+0.3	5.0	29.8	−24.8	1.0	0.1	+0.9	+2		+0.7
3*	−6.5	98.4	−105.0	−1.0	2.2	−3.2	−2.2	18.8	−21.0	0.0	0.6	−0.6	+4.5		+1.6
4	−2.0	40.8	−42.8	0.8	3.1	−2.4	−0.5	11.7	−12.2	0.3	0.4	−0.2	+0.7		+0.3
5	3.6	−117.6	+121.2	1.7	−7.0	+8.7	1.9	−10.6	+12.4	0.1	−0.5	+0.6	+3.0		+1.2
6	−3.8	−3.3	−0.5	−0.3	1.2	−1.5	0.2	−1.8	+2.0	0.0	−0.1	+0.1	+2.1		+0.9
7	9.4	59.2	−49.8	2.0	0.5	+1.6	2.6	16.0	−13.5	0.2	0.3	−0.1	+2.1		+0.8

Areas under the curve (AUC) were computed by calculating the sum of changes in androgens from baseline to days 7, 14, 21, and 28. The absolute differences between AUCs were calculated by subtracting the value without anakinra from the value with anakinra, i.e., negative values indicate a reduction upon anakinra. Patient numbers do not correspond to real patient identification numbers. Patients taking metformin are marked in gray and the one patient taking progesterone is marked with an asterisk.

AUC, area under the curve; BMI, body mass index.

**Figure 3:**
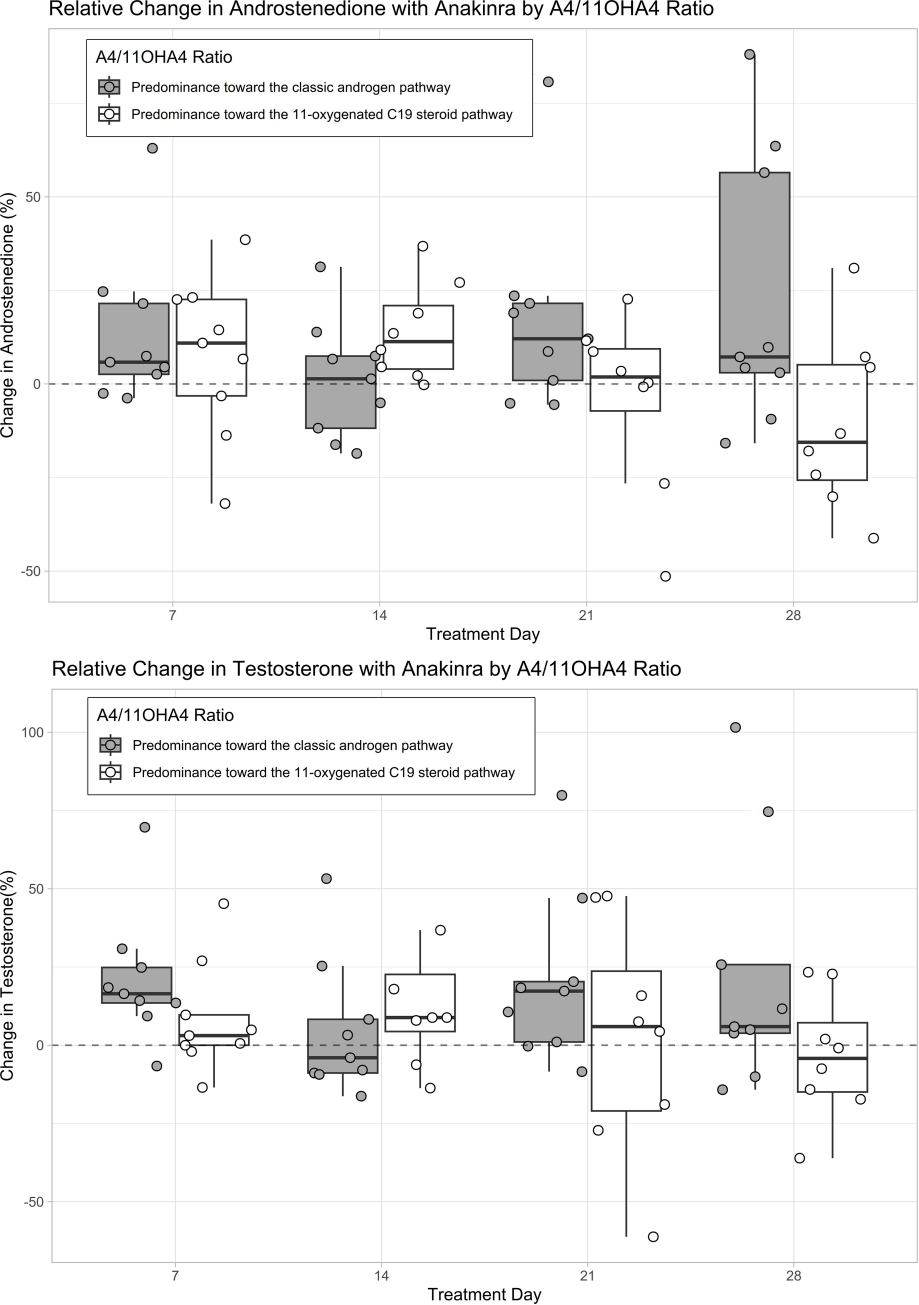
Absolute changes in androgens with anakinra based on the predominant androgen pathway. The predominant androgen pathway was computed by dividing baseline androstenedione (A4) levels (classic androgen pathway) by baseline 11β-hydroxyandrostenedione (11OHA4) levels (11-oxygenated C19 steroid pathway) (42). A quotient above the median (0.872 nmol/nmol) was considered as a predominance toward the classic androgen pathway and a quotient below the median was considered as a predominance toward the 11-oxygenated C19 steroid pathway. The boxplots represent relative changes in androgens from baseline. Values above the dashed line represent an increase and values below the dashed line represent a decrease in androgens as compared to baseline. Androgen levels were measured with tandem-mass spectrometry (LC-MS/MS).

We observe a tendency toward a reduction in DHEAS but not in DHEA, 11OHA4, 11OHT, 11KA4, or 11KT upon anakinra (**Supplementary Figure S5**). The effect of anakinra on testosterone and androstenedione appeared to be more pronounced in the nine women with an androstenedione-to-11OHA4 ratio below the median than in the nine women with a ratio above the median (**Figure 3**).

## 4 Discussion

This study has three main findings. First, we observed an increase in androgen levels over both 5-week study periods, which tended to be mitigated by IL-1 antagonism with anakinra, especially toward the end of treatment. Second, menstrual bleeding occurred in 27% (5/18) of the patients by the end of treatment with anakinra, of which 60% (3/5) were previously oligo-/amenorrhoeic and of which 80% (4/5) were considered to be ovulatory based on the observation of a peak in progesterone. Third, anakinra significantly reduced CRP levels but did not reduce absolute androgen levels compared to baseline.

The primary objective was to investigate whether IL-1 receptor antagonism ameliorates hyperandrogenemia in patients with PCOS. Testosterone and its precursor androstenedione are the primary androgens synthesized by ovarian theca cells (31–33). Recent data suggest that serum androstenedione is superior to testosterone in the assessment of hyperandrogenemia and indicator of metabolic risk in patients with PCOS (34–38) although guidelines primarily recommend measurement of serum free or total testosterone levels to assess hyperandrogenemia in patients with suspected PCOS (39). Contrary to our hypothesis, we observed an overall tendency toward an increase in androgen levels during the 28-day treatment with IL-1 receptor antagonist anakinra, which reached significance 7 days after treatment start. Because of these surprising findings, we conducted a sub-study to measure androgen levels in the same cohort but without anakinra. When comparing the cumulative changes in androgens with and without anakinra, anakinra appeared to have mitigated the increased androgen exposure in four of six patients. In line with this interpretation, it was recently shown that the follicular fluid of women with PCOS contains higher levels of IL1-β and IL-18 and displays an activation of the NF-kB pathway as compared to controls (40). Accordingly, beneficial effects of anti-inflammatory treatment with an NFκB inhibitor on ovarian steroid metabolism and ovulation in patients with PCOS were demonstrated (24). Interestingly, a cross-sectional study suggested that combined oral contraceptive pills (OCPs) upregulate proinflammatory genes in women with PCOS (41), so that further trials should also include women with PCOS and OCP since they might even have greater benefit from anti-inflammatory treatment. In contrast, a recent *in vitro* study demonstrated that exposing human granular ovarian cells to metformin reduced IL-1β and pyroptosis (42), which advocates an anti-inflammatory role for metformin. A randomized trial in 40 PCOS women investigating the effect of a 12-week treatment with metformin or metformin plus aerobic exercise suggests that physical activity might potentiate the anti-inflammatory effect of metformin (43). However, metformin only partially acts through the IL-1 pathway (44, 45); therefore, a concurrent targeted IL-1 inhibition would have a synergistic effect. Previously performed clinical trials with IL-1 blockers indicated the potential beneficial effect not only on prediabetes and diabetes, but also on cardiovascular morbidity and mortality (46–48). Therefore, blocking IL-1 could be a promising holistic new treatment option in women with PCOS. Whether this could be best achieved with anakinra, metformin, or exercise, alone or in combination, should be investigated in further trials.

Recent data suggest that the classic androgens testosterone and androstenedione are not the only components of hyperandrogenemia in PCOS. A cross-sectional study showed that women with PCOS have increased 11-oxygenated androgen levels and that these adrenal androgens represent the main circulating androgens and a greater proportion of total androgens as compared to control women (49). Because we did not observe a reduction in the adrenal 11-oxygenated androgens, we suspect the effect of anakinra on hyperandrogenemia to take place at the ovarian level. However, the effect of anakinra on testosterone and androstenedione seemed to be more pronounced in women with a relative predominance toward the 11-oxygenated C19 steroid pathway indicated by a lower androstenedione-to-11OHA4 ratio. Whether this distinction is artificial or has pathophysiological and potential therapeutic implications needs further research.

Five patients reported menstrual bleeding during anakinra treatment, of whom three previously suffered from oligo-/amenorrhea, which suggests that IL-1 antagonism may promote more regular cycles. In addition, because of the observed peak in progesterone, most of these cycles were thought to be ovulatory. In comparison, no progesterone peak was observed in the two menstruating patients during the observational phase, although one ovulatory cycle might have been missed due to the limited follow-up duration. Overall, this indicates potential for the use of anakinra in enhancing fertility in women with PCOS (50–52). However, we only observed a single menstrual cycle, and therefore, further trials with extended intervention periods (e.g., 3–6 months) are necessary to conclusively determine the effect of anakinra on the hypothalamic–pituitary–ovarian axis.

The observed CRP reduction upon anakinra underpins that chronic low-grade inflammation in PCOS is, at least partially, driven by the proinflammatory cytokine IL-1. Although the anti-inflammatory effect was visible already by day 7, CRP levels returned to baseline levels on day 14 because of injection site reactions. In accordance with the literature, injection site reactions were temporary, though still present at the end of treatment in 76% of the patients (53). Anakinra is known to induce injection site reactions in up to 70% of patients in the first weeks of treatment (53, 54), which can severely impair drug efficacy depending on their severities (55). As injection site reactions were most pronounced on days 10 to 15, and faded with time, CRP levels correspondingly decreased on days 21 and 28, but to a lesser extent than the initial reduction on day 7. CRP levels returned to baseline 1 week after treatment. The blunted CRP reduction might explain why we found no association between changes in CRP and changes in androgens that would further support an interplay between chronic inflammation and androgenesis. Since CRP synthesis in the liver is also simulated by cytokines other than IL-1, the reactive increase in CRP does not invalidate IL-1 antagonism at the hepatic and, more importantly, ovarian levels. These other cytokines released by the injection site reactions were not antagonized by anakinra and therefore might still have mitigated the anti-inflammatory effect of anakinra on the increase in androgens. Without anakinra, CRP tended to decrease on days 7 and 14, which is in line with observations in healthy women and overweight women without PCOS (56).

Some concerns about a long-term IL-1 receptor antagonism might be raised as IL-1α and IL-1β are important proinflammatory mediators. However, they are not crucial for pathogen elimination. Two systematic reviews and meta-analyses of ~3,000 patients showed that risk of infections was not significantly increased for anakinra compared with placebo (54, 57). In our study, all patients experienced injection site reactions. These injection site reactions are harmless and transient and occur at the beginning of anakinra therapy. Informing patients before treatment start improves therapeutic adherence, as seen in our study.

Our studies have limitations. First, the observational sub-study took place 1 year after the interventional study and included only a part of the initial cohort; therefore, only a small sample size was available to compare both studies and one patient newly took gestagen during the last 2 weeks of the observational study. Additionally, most patients gained some weight, which, along with older age and undergoing fertility treatments, could have influenced androgenemia. A double-blind placebo-controlled crossover design would have represented a more appropriate design. Second, patients only received anakinra for 28 days. A longer observation period would have reduced the impact of the CRP increase secondary to the injection site reactions, which only occurred at the early stage of treatment and would have also allowed us to study the impact on ovulation and menstrual periods over a longer period. The strength of the study relies on the inclusion of patients with PCOS with biochemically confirmed hyperandrogenemia and elevated inflammatory markers at baseline, and on the measurement of androgens with the gold standard technique LC-MS/MS in an experienced laboratory.

In summary, this is the first study data on the use of IL-1 receptor antagonist in women with PCOS. IL-1 antagonism with anakinra, as compared to no treatment in a subsequent observational study, may have the potential to mitigate androgen exposure in PCOS, with our results limited by the constraints of study design and sample size. We recorded the onset of menses in five previously oligomenorrheic patients. Future studies should further investigate the effect of a long-term inflammatory blockade of the IL-1 system, using long-acting drugs (antibodies) or oral agents (inflammasome inhibitors) on clinical and biochemical hyperandrogenism and cardiovascular comorbidities in women with PCOS.

## Data Availability

The datasets presented in this article are not readily available because of patient confidentiality. Deidentified individual participant data will be shared upon publication to researchers who provide a methodologically sound proposal to achieve the aims in the approved proposal. Proposals should be directed to the corresponding author. To gain access, data requestors will need to sign a data access agreement. Requests to access the datasets should be directed to MC-C, mirjam.christ-crain@usb.ch.
