# Supplementary material for: Effects of interleukin-1 receptor antagonism in women with polycystic ovary syndrome—the FertIL trial
Source: Front Endocrinol (Lausanne). 2024 Sep 11;15:1435698. doi: 10.3389/fendo.2024.1435698 (PMC11423739; doi:10.3389/fendo.2024.1435698)

SUPPLEMENTARY MATERIAL

-

Effects of Interleukin 1 Receptor Antagonism in Women with Polycystic Ovary Syndrome – The FertIL Trial

TABLE OF CONTENT

Detailed Statistical Methods 2

Supplementary Table S1 Baseline Characteristics...3

Supplementary Table S2. Laboratory Values Of Patients Who Participated In The Main Interventional Study And The Observational Sub-Study 5

Supplementary Figure S1 Absolute Changes In Androgens Upon Anakinra.................................6

Supplementary Figure S2 Time Course Of Androgens And CRP Levels 7

Supplementary Figure S3 Progesterone Levels With and Without Anakinra 8

Supplementary Figure S4 Estradiol, FSH, LH And AMH Levels Upon Anakinra 9

Supplementary Figure S5 Absolute Changes In Gonadal And Adrenal Androgens With And Without Anakinra 10

Detailed Statistical Methods

All analyses were performed using the statistical program R (version 4.2.3 or higher). Baseline characteristics were summarized using descriptive statistics. A two-sided significance level of 0.05 was used for every analysis except for interaction terms of linear mixed models for which we considered a p-value < 0.1 as justification for subgroup analyses. P-values were not adjusted for multiple testing.

The primary endpoint, the change in fasting serum androstenedione levels from baseline to day 7, was tested with a paired Wilcoxon signed-rank test.

Subgroup effects for the primary endpoint were evaluated in the interventional dataset only, by fitting linear regression models with log transformed androgen values during treatment as outcome variable, baseline androgen values as covariate, and subgroup and their interaction term with the covariate as predictors. The following predefined subgroups were examined: CRP below or above the median at baseline, presence of injection site reactions (yes/no) on day 28, CRP reduction (yes/no) defined as change in CRP levels from baseline to day 28 < 0 mg/l), ovulation between baseline and day 35 (yes/no), menstruation after the start of treatment (yes/no), amenorrhea versus oligo-/normomenorrhea at baseline.

For the statistical analysis of steroid results, in case < 25% of the values of a given steroid were below the lower limit of quantification (LLOQ) or undetectable, these values were replaced with the mean of 0 and the LLOQ for statistical analysis. In case these values represented > 25% of all the values of a given steroid, a random value between 0 and LLOQ was imputed for statistical analysis. Unchanged measured values were kept unchanged for graphical representation.

Androstenedione, testosterone, and CRP values at each visit were compared to baseline values with a paired Wilcoxon signed-rank test in both the interventional and observational datasets. The area under the curve (AUC) of changes in androgens between day 0 and day 28 with and without anakinra were computed for each patient who participated in the interventional and in the observational study.

Additional androgens (11OHA4, 11OHT, 11KA4, 11KT, DHEA and DHEAS) were compared graphically with boxplots. The predominant origin of androgens (ovarian or adrenal) was evaluated by dividing by baseline androstenedione by 11OHA4 and comparing the response to anakinra between participants with a quotient below versus above the median.

Supplementary Table S1 Baseline Characteristics

Baseline characteristics of the 18 study participants included in the intervention study with anakinra and the sub-cohort (n = 7) included in the observational study. Continuous variables are presented as means (SD) or medians [IQR] depending on data distribution. Categorical variables are presented as counts (%).

Variable	N = 18	N = 7
Age, years	27 [23, 32]	35 [30, 36]
European ancestry, n (%)	16 (89)	
Alcohol consumption, glasses per week	0.7 (1.2)	
Active smoking, n (%)	6 (33.3)	
Diabetes mellitus, n (%)		
Type 1	1 (5.6)	
Type 2	1 (5.6)	
Arterial hypertension	0 (0)	1 (14)
Diagnosed after the intervention study		1 (14)
History of psychiatric disease, n (%)	4 (22)	2 (28)
Diagnosed after the intervention study		2 (28)
Medication, n (%)		
Metformin	4 (22)	3 (42)
Started after the intervention study		1 (14)
Insulin	1 (6)	
GLP-1 receptor agonist	1 (6)	0 (0)
Antidepressant drugs	2 (11)	
Oral progesterone		1 (14)
Started after the intervention study		1 (14)
Menstrual dysfunction, n (%)		
Amenorrhea	9 (50)	
Oligomenorrhoea	7 (39)	
Time since the onset of the last menstruation on inclusion day, days		3 [3, 4]
Number of menstrual cycles, n		
12 months prior inclusion		9 [6, 10]
6 months prior inclusion		3 [2, 6]
3 months prior inclusion		1 [1, 3]

Fertility, n (%)		
History of infertility*	5 (63)	
History of pregnancy loss*	4 (50)	
Try to get pregnant since the interventional study		3 (43)
No pregnancy		2 (29)
Spontaneous abortion		1 (14)
Clinical Examination		
Systolic blood pressure, mmHg	115 (10)	
Diastolic blood pressure, mmHg	80 [74, 82]	
Heart rate, bpm	73 [70, 78]	
Height, cm	164.0 [159.8, 168.8]	
Weight, kg	88.2 (21.8)	74.5 [66.7, 113.0]
Weight change since the interventional study (kg)		2.1 [2.1, 4.0]
Weight change since the interventional study (%)		3.1 [2.3, 5.1]
Waist circumference, cm	99.0 [89.3, 105.0]	100.0 [78.5, 113.0]
Hip circumference, cm	111.8 (12.2)	
Waist-hip ratio	0.9 [0.8, 0.9]	
BMI, kg/m^2	32.6 [28.8, 37.3]	31.4 [25.6, 40.4]
Ferriman Gallwey score	7 [1-12]	
Presence of acne, n (%)	13 (72)	
Acne score in patients with acne	3 (2, 5)	
Sebum production, $\mu g/cm^2$	128 [92.3, 184.0]	
Alopecia, n (%)	1 (5.6)	
Acanthosis nigricans, n (%)	3 (16.7)	
PCO Morphology, n (%)	14 (78)	

BMI = Body Mass Index,GLP-1 = glucagon-like peptide 1 ,PCO = Poly Cystic Ovaries.

*of n=8 patients who tried to conceive

Supplementary Table S2. Laboratory Values Of Patients Who Participated In The Main Interventional Study And The Observational Sub-Study

Values are represented as median and interquartile range [IQR]. Androgen levels were measured with tandem-mass spectrometry (LC-MS/MS). High-sensitivity CRP was measured with an immunoturbidimetric assay (Tina-quant C-Reactive Protein IV; Roche Diagnostics GmbH). Oestrogen, FSH, LH, prolactin and SHBG were measured with ECLIA (Elecsys Estradiol III, Elecsys FSH, Elecsys LH, Elecsys SHBG, Roche Diagnostics GmbH).

Treatment	Anakinra					No treatment				
Study Day	Baseline	7	14	21	28	Baseline	7	14	21	28
n	7	7	7	7	7	7	7	6	7	6
CRP, mg/l	2.0 [1.4, 2.7]	1.5 [0.9, 2.5]	1.6 [1.0, 11.3]	1.50 [0.9, 2.0]	1.1 [1.0, 2.2]	2.1 [1.9, 10.3]	1.9 [1.3, 2.5]	2.4 [1.0, 5.7]	1.9 [1.5, 2.8]	2.0 [1.4, 2.5]
Oestrogen, pmol/l	227.0 [218.5, 254.5]	248.0 [213.5, 284.0]	235.0 [221.0, 342.0]	248.0 [215.5, 409.0]	242.0 [223.0, 402.0]	228.0 [156.0, 261.0]	247.0 [202.0, 264.5]	258.0 [246.5, 280.0]	248.0 [239.0, 317.5]	228.0 [204.3, 287.0]
FSH, IU/l	6.2 [5.2, 6.9]	6.2 [6.0, 6.8]	5.7 [5.2, 6.5]	6.2 [2.6, 6.5]	6.1 [4.1, 6.5]	6.1 [4.5, 6.9]	6.3 [5.1, 6.9]	6.2 [5.3, 6.4]	5.6 [4.9, 5.8]	5.5 [4.9, 5.6]
LH, IU/l	11.5 [10.5, 19.6]	14.8 [11.5, 17.8]	15.1 [9.5, 19.6]	12.3 [10.1, 14.9]	10.7 [9.6, 15.6]	11.1 [9.4, 12.7]	12.3 [9.7, 13.3]	11.3 [10.1, 17.0]	11.6 [11.2, 17.6]	11.5 [10.4, 12.1]
SHBG, nmol/l	40.1 [27.1, 48.0]	39.9 [29.2, 50.4]	37.5 [24.0, 50.9]	41.0 [28.9, 49.1]	35.6 [28.6, 55.5]	31.6 [26.7, 55.5]	33.1 [25.2, 49.8]	39.3 [25.6, 47.4]	30.1 [25.4, 46.9]	30.1 [22.8, 56.2]
Androstenedione, nmol/l	8.6 [7.0, 9.6]	8.4 [6.6, 11.0]	9.4 [7.3, 10.9]	8.7 [6.3, 10.1]	7.2 [5.8, 11.3]	11.4 [8.3, 12.4]	12.3 [6.6, 13.5]	10.9 [7.5, 12.8]	11.9 [7.0, 14.4]	13.3 [8.2, 14.5]
Testosterone, nmol/l	1.6 [1.5, 2.1]	1.9 [1.6, 2.4]	1.7 [1.5, 2.4]	2.0 [1.8, 2.4]	2.0 [1.6, 2.4]	2.4 [1.6, 2.8]	2.1 [1.9, 2.9]	2.2 [2.0, 2.5]	2.2 [1.9, 3.4]	2.3 [2.0, 3.2]
CRP = C reactive protein, FSH = Follicle stimulating hormone, LH = luteinizing hormone, n.a. = not applicable, SHBG = sex hormone binding globulin.										

Supplementary Figure S1 Absolute Changes In Androgens Upon Anakinra

The boxplots represent changes in androgens from baseline, which were computing by subtracting the baseline androgen concentration from the corresponding follow-up value. Values above the dashed line represent an increase and values below the dashed line represent a decrease in androgens as compared to baseline. Androgen levels were measured with tandem-mass spectrometry (LC-MS/MS).

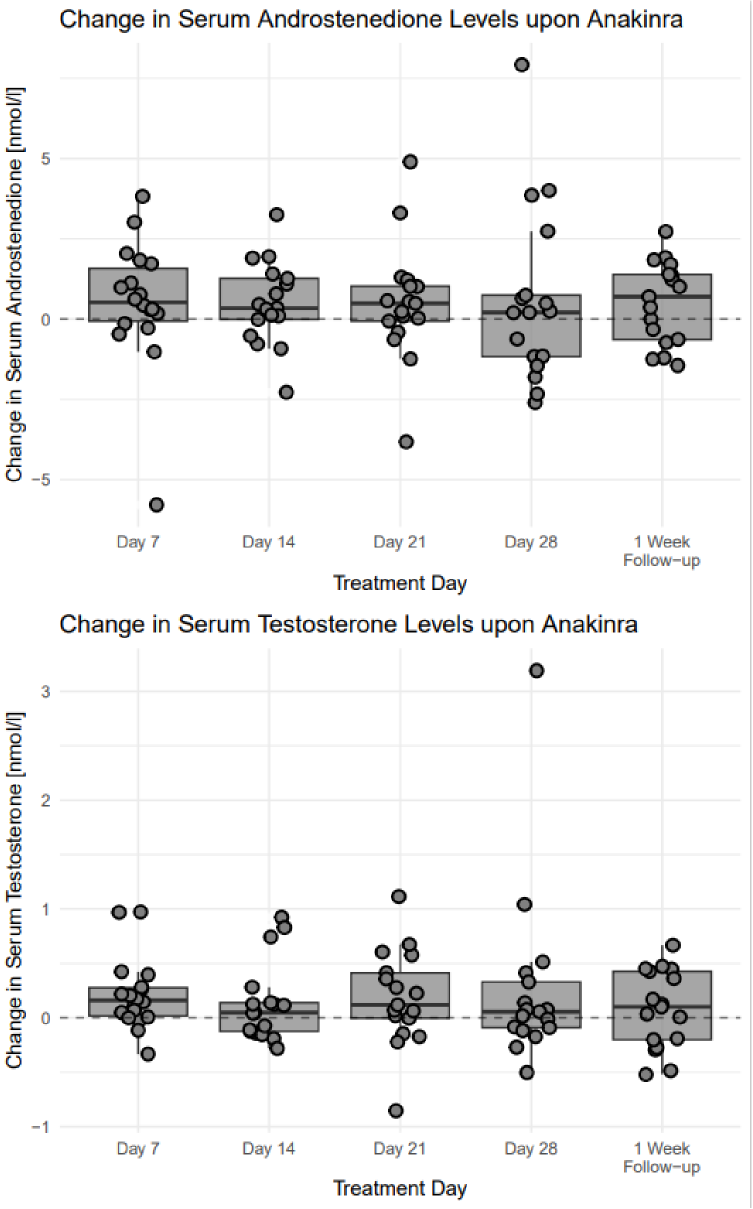

Supplementary Figure S2 Time Course Of Androgens And CRP Levels

Boxplots show serum androstenedione, serum testosterone levels and CRP during treatment with anakinra (n = 18). Androgen levels were measured with tandem-mass spectrometry (LC-MS/MS). High-sensitivity CRP was measured with an immunoturbidimetric assay (Tina-quant C-Reactive Protein IV; Roche Diagnostics GmbH).

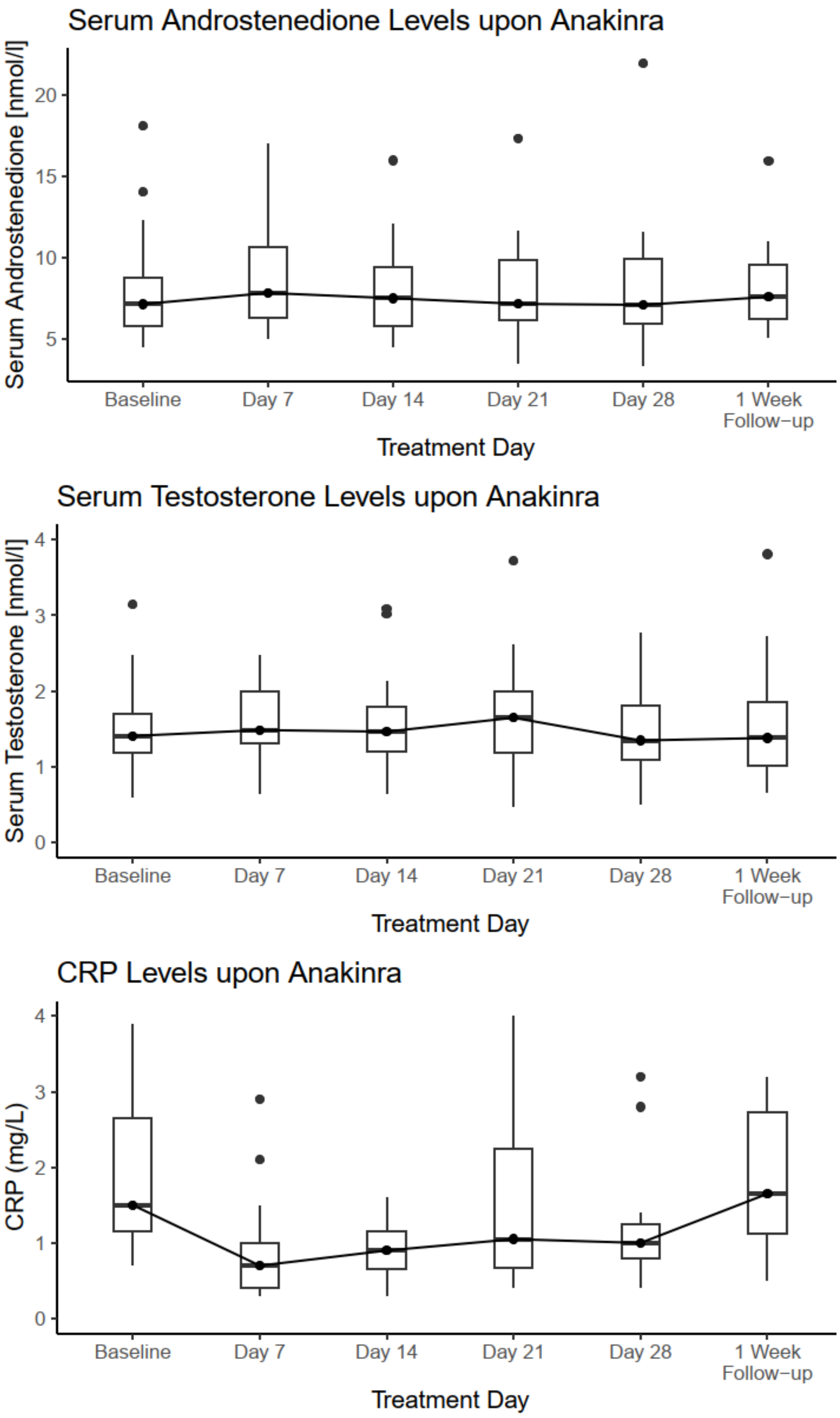

Supplementary Figure S3 Progesterone Levels With and Without Anakinra

Progesterone was measured with tandem-mass spectrometry (LC-MS/MS). Each line represents an individual patient. (A) Four out of 18 patients experienced a peak in progesterone upon anakinra, including 2 of the 4 patients taking metformin ($^{+}$). Three of the 4 patients passed the progesterone cut-off of ≥ 5.8 nmol/l indicative for ovulation. (B) In patients who also participated in the observational phase, 2 out of 7 patients experienced a peak in progesterone upon anakinra, 1 of which was above the cut-off of ≥ 5.8 nmol/l indicative for ovulation (showed with a dashed line). (C) Two out of 7 patients experienced a peak in progesterone without anakinra, 1 of which upon oral progestin (*).

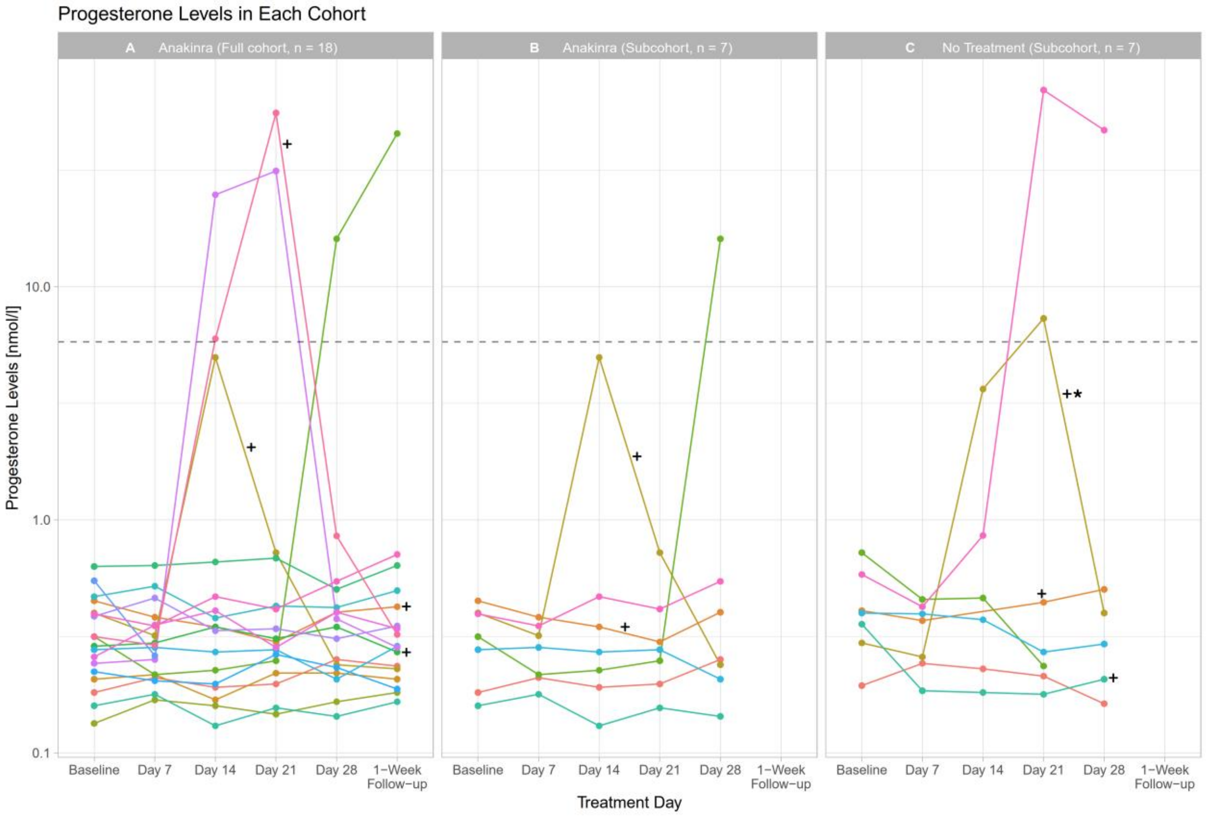

Supplementary Figure S4 Estradiol, FSH, LH And AMH Levels Upon Anakinra
Boxplots show absolute serum oestradiol, FSH, LH and AMH levels during anakinra treatment (n = 18). One outlier on day 14 and one outlier on day 28 are not represented in the oestradiol plot. AMH, estradiol, FSH, LH, were measured with ECLIA ((Elecsys AMH Plus, and Elecsys Estradiol III, Elecsys FSH, Elecsys LH, Roche Diagnostics GmbH). Abbreviations: AMH = anti-mullerian hormone, FSH = Follicle stimulating hormone, LH = luteinizing hormone.

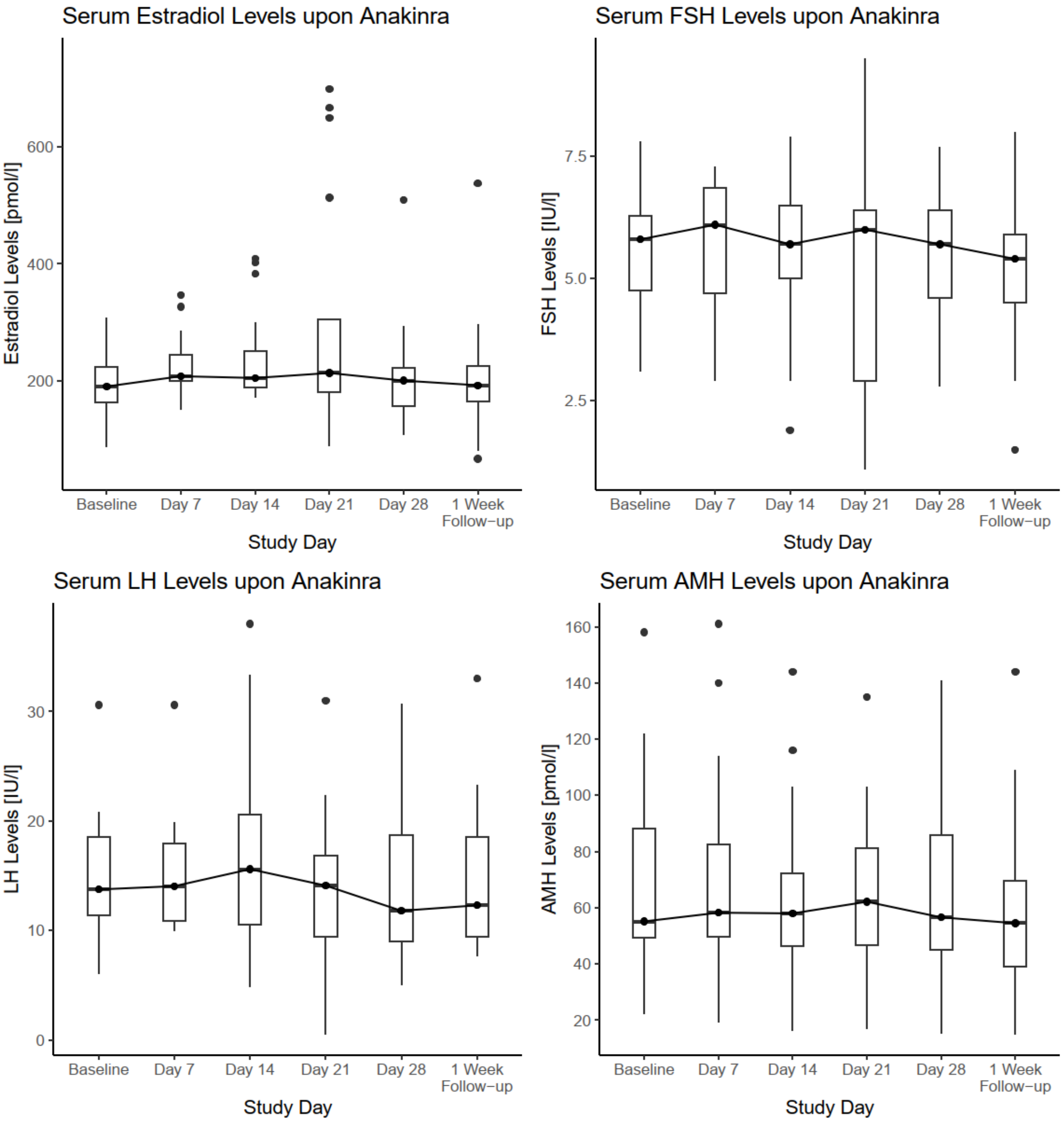

Supplementary Figure S5 Absolute Changes In Gonadal And Adrenal Androgens With And Without Anakinra

The boxplots represent relative changes in androgens from baseline. Values above the dashed line represent an increase and values below the dashed line represent a decrease in androgens as compared to baseline. Androgen levels were measured with tandem-mass spectrometry (LC-MS/MS). 11OHA4 = 11β-hydroxyandrostenedione, 11OHT = 11β-hydroxytestosterone, 11KA4 = 11-ketoandrostenedione, 11KT = 11-ketotestosterone, DHEA = dehydroepiandrosterone, DHEAS = dehydroepiandrosterone sulfate.

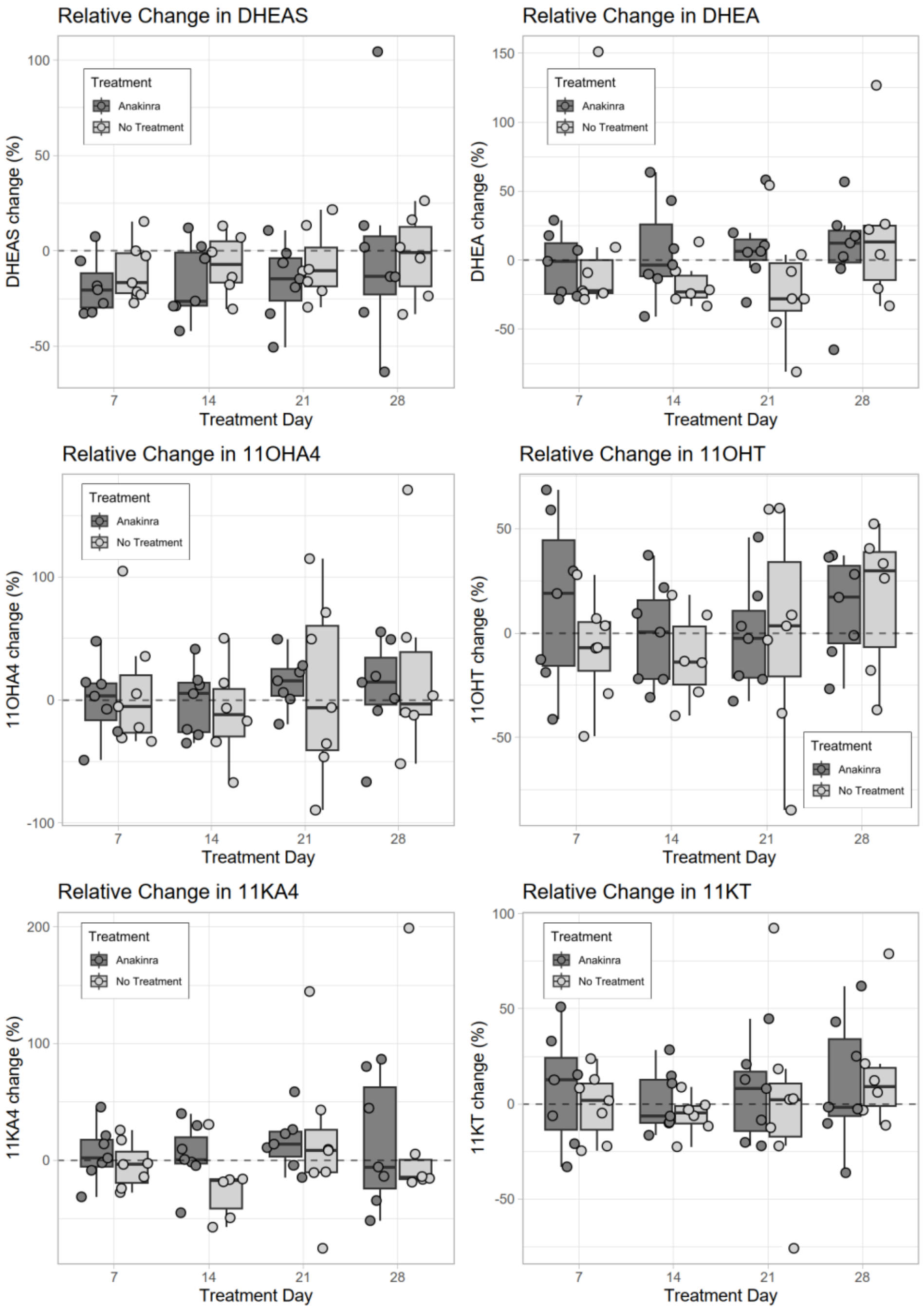

Supplement: Supplementary file 1 [file DataSheet1.pdf]
